# A Mathematical Model of Statin Anti-Hyperlipidemic Drug Reactivity and Diverse Concentrations of Risk Toxicity

**DOI:** 10.3390/jcm14072331

**Published:** 2025-03-28

**Authors:** Evangelos Mylonas, Christina Mamareli, Michael Filippakis, Ioannis Mamarelis, Jane Anastassopoulou, Theophile Theophanides

**Affiliations:** 1 Department of Digital Systems, University of Piraeus, 18534 Piraeus, Greece; evanmylonas7@gmail.com (E.M.); mfilip@unipi.gr (M.F.); 2 Athens Institute for Education and Research, 10677 Athens, Greece; christinamma32@yahoo.gr (C.M.); i.anastassopoulou@gmail.com (J.A.); 3 Cardiology Department, 401 Military General Hospital, 11527 Athens, Greece; imamarelis@gmail.com

**Keywords:** statins, mathematical model, fractional order Caputo hypercholesterolemia, atherosclerosis, oxidative stress, FTIR spectroscopy

## Abstract

**Background/Objectives:** Statins decrease the risk of cardiovascular events by lowering low-density lipoproteins (LDLs). Despite this, statins induce toxic effects by a mechanism of action that has not yet been elucidated. The aim of the present work was to create a mathematical simulation model to evaluate the effect of statins on LDL concentration reduction and the threshold value of toxic reversible concentrations. **Methods:** Fifteen calcified coronary artery biopsies from non-diabetic hyperlipidemic patients treated with statins were used. For this study, an advanced modified model including the Caputo Fractional Operator and molecular dynamics was employed. **Results:** The new characteristic absorption bands in the FTIR spectral region frequencies near 1744 and 976 cm^−1^, assigned to the chemical functional groups of aldehydes (vCHO) and phosphates V(PO_4_^3−^) of the atheromatic plaques, respectively, were used for mathematical model development. The energy of the functional chemical bonds caused by redox modifications during atheromatic plaque progression was used to show the effects of statin concentrations numerically. The model provides the anti-atheromatic effects of statins by the inhibition of LDL formation. Furthermore, the mathematical model highlights the dose medication–statin dependence on the reverse point of the statins’ protective role. **Conclusions:** The new mathematical model shows both the beneficial and harmful actions of statins, which are associated with critical dose-dependent treatments with statins. The model also indicates that, upon increasing the statin dose, excessive secondary oxidation products were obtained. These products control the upregulation of the biological response by triggering other new pathways of redox homeostasis reactions.

## 1. Introduction

There is a growing body of scientific papers regarding clinical and animal models that suggest hyperlipidemia remains one of the most common problems globally and is strongly associated with the atherosclerosis of coronary arteries and other cardiovascular events [1,2]. Hyperlipidemia is a long-term process occurring through the peroxidation of polyunsaturated fatty acids during the deregulation of cell redox potential, caused by free radical biochemical reactions supported by oxidative stress from both endogenous and exogenous sources. The excess of free radicals, caused by oxidative stress, leads to elevated serum low-density lipoprotein (LDL) concentrations [3]. It has been documented that, under pathological conditions, the accumulation of LDLs and other advanced peroxidation end products (AGEs) is related to the progression and regression of atheromatic plaque formation, triggering the secretion of chemokines which promote other pathological pathways [4,5]. Therefore, the main goal of the people working in the field is to develop new anti-hypercholesterolemic drugs to minimize the side effects. Actually, statins, which are hydroxymethyl glutaryl coenzyme A reductase inhibitors, are the most widely prescribed drugs globally and have shown promising potential for the prevention of cardiovascular events in high-risk patients. Moreover, statins have shown antioxidant and anti-inflammatory properties [6]. It has been demonstrated that more significant reduction in LDL levels is related to the dose of statin medications. However, it was shown that for patients with multiple medical co-morbidities, statins show adverse reactions associated with dose–effect, although the mechanism of this remains challenging to elucidate [7,8]. Moreover, there is no clear threshold of LDL dependence on statin medication for all patients. Our research focuses on lowering cholesterol and the risk of cardiovascular events in order to reduce the side effects caused by statins in some patients.

In modern clinical applications, quantitative analytical tests play a pivotal role in monitoring and detecting the development of the disease. The existing analytical methods provide no answers regarding the conformational changes at a molecular level. Fourier-Transform Infrared (FTIR) spectroscopy is a sensitive, non-destructive, and easily applicable technique for the detection of differences between “diseased” and “healthy” tissues or fluids by their characteristic spectral bands [4,5]. The method is based on the interaction between infrared rays and the tissues of the aortic valve and carotid or coronary arteries, which give rise to vibrational frequencies obtained via the harmonic and anharmonic energy levels of the molecules that form the atheromatic plaque. In reality, infrared spectra are associated with the length, strength constant, and energy of the chemical bonds of the molecules and the environment where the molecules vibrate. Infrared spectra are not simple and are consistent with all vibrational absorption bands that arise from the tissue components, and they are characteristic due to the various functional chemical groups of biomolecules, such as the groups NH, COO^−^, C=O, C-O-C, PO_2_^−^, and PO_4_^3−^ of proteins, amino acids, glycosides, DNA, or hydroxyapatite, which always appear to have approximately the same energy in wavenumbers (cm^−1^) [4,5,9,10]. Thus, spectroscopy gives us the ability to gain information on the molecular structures of biological molecules that could be associated with the disease. The coupling of FTIR spectrometers with an ATR (Attenuated Total Reflection) apparatus has led to the successful, simultaneous recording of all the components of non-homogenous tissues and cells and requires small amounts of a sample (even thin sections of 5−10 μm) without any special preparation.

In recent decades, the increasing speed and capacity of computers have allowed scientists to combine complex clinical data and develop new software and mathematical models to approach the pathways of atheromatic plaque formation and progression [9,10]. Some recent mathematical models refer to the study of atherosclerotic plaque development [11,12,13]. Today’s existing mathematical models are based on steady-state (rigid) atherosclerotic plaques and do not include the molecular dynamics of atherosclerosis products, which are affected by time and medication. Rather than the above mathematical models, the present research focuses on using fractional models, which could be used to reveal improved outcomes in prevention, diagnosis, and therapeutic approaches since these have not yet been established. For this purpose, it is important to develop a mathematical model that will predict the anti-hyperlipidemic role of statins in LDL development. Finally, we focus on developing an advanced modified model based on a Fractional Order Calculus equation set to estimate and manage the concentration of statins in patients to optimize and emphasize the adverse critical point.

The design of the mathematical model was based on the analysis of the FTIR spectral absorption bands and the free energy of the functional groups of the most important and clinically recognized products in the atheromatous plaques of the coronary arteries. It is known that the pathogenesis of atherosclerosis is a multifunctional process associated with age, hypertension, hyperlipidemia, diabetes, chronic inflammation, and other risk factors and diets, but the mechanism of atherogenesis is not yet well known.

## 2. Materials and Methods

FTIR spectroscopy: The FTIR spectra were of 15 calcified coronary artery biopsies from non-diabetic hypercholesterolemia patients (aged 54–85 years) who had undergone coronary endarterectomy during Coronary Artery Bypass Graft (CABG) surgery and been treated with statins. The samples were fixed in formalin solutions immediately after removal as described elsewhere [4,5]. The FTIR spectra were recorded with a Nicolet 6700 spectrometer (Waltham, MA, USA) equipped with a diamond-based ATR-FT-IR accessory. By using this technique, tissues are not homogenized, and we may study different parts of the tissue (by mapping). To obtain good-quality infrared spectra, sufficient small quantities of tissues with a depth of 10 μm up to 1 mm were used. To increase the quality of the spectra, each spectrum consisted of 120 co-added spectra at a spectral resolution of 4 cm^−1^, and the OMNIC 7.2a software was used for data analysis as described elsewhere [4,5,9]. The specimens were fixed in formalin but were not incubated in paraffin. It was observed that by using hexane to extract the paraffin, important molecules that are also soluble in hexane were simultaneously removed from the tissues. The obtained spectra from atheromatic coronary arteries were compared between each patient and the corresponding spectra of healthy tissues. Since FTIR spectroscopy is based only on the energy of the chemical bonds of the molecules, the number of 15 patients was considered sufficient in this case.

Ethics: The study was designed according to the Declaration of Helsinki, Greek ethical rules, and the permission of the Scientific Board of the 401 Military Hospital (No. 07/2907/2024). The samples were taken after removal from the patients for ex vivo study.

### Mathematical Model

For the mathematical model, the clinical serum LDL and HDL levels of the patients and the frequency of the absorption bands of the characteristic functional groups were considered. A modified model based on the above parameters, including the Fractional Caputo Derivatives, was developed to estimate the effect of anti-hypercholesterolemia medication on patients. Our mathematical calculations were evaluated using MATLAB software R2024b. The numerical solution to our problem was based on the Predictor–Corrector (PECE) method developed by Garrappa [14], an algorithm based on the fractional form of the Adams–Moulton method for fractional differential equations (FDEs).

## 3. Results

### 3.1. FTIR Spectroscopic Data

In infrared spectroscopy, each spectral band of a molecule is characteristic and always appears at the same characteristic wavenumber (cm^−1^) in its unique fingerprint spectrum. The exact positions of the bands give information on the energy of the bond and electron-withdrawing or -donating strength in the intra- and inter-molecular environments in which the molecule is vibrating [4,5,9]. Any fluctuation of the main infrared position of the characteristic band of a chemical bond can be rationalized based on structural changes induced by the disease. Table 1 displays the most important characteristic FTIR spectral vibrational absorption bands of the tissues, the assignments, and the free energy of the corresponding functional chemical bonds of the atherosclerotic coronary artery components produced by the disease. The energy of the chemical bonds is directly proportional to wavenumbers (E = hcW, h = Planck’s constant, c = velocity of light, W = wavenumber in cm^−1^, 1 cm^−1^ = 2.85914 × 10^−3^ kcal/mol). As shown in Table 1, higher wavenumbers have higher energy than lower wavenumbers.

For simplicity in our research, we divided the atheromatic components into the following two phases: the organic phase, which contains lipids, proteins, glucosides, and other organic molecules; and the inorganic phase, which contains inorganic salts and organometallic molecules, which are obtained from calcified proteins, biological-like hydroxyapatites, and minerals such as inorganic hydroxyapatite and calcium carbonates.

### 3.2. Organic Phase of Atherosclerotic Plaque–Oxidative Stress Interaction

This particular organic phase contains the peroxidation end products that have resulted from membrane lipid–free radical interactions, named foam cells. It is well documented that malondialdehyde (MDA, CHO-CH_2_-CHO) is one of the most important diagnostic sub-products of non-enzymatic lipid peroxidation in cardiovascular diseases upon oxidative stress development [9,15,16]. This is easily recognizable by thiobarbituric acid assay. Among the recorded FTIR spectra from the present research, MDA is detected as the intense stretching vibration band of the functional group *v*CHO which appears at 1744 cm^−1^ [4,5,9]. The intensity height of this vibrational band is very sensitive to serum LDL concentration and is reduced with statin medication [4,5,9]. This band is also indirect evidence that statins could act as anti-inflammatory agents or as free radical scavenger receptors.

### 3.3. Mathematical Model

LDL is the most important “bad” lipoprotein related to blood cholesterol accumulation and the atherosclerosis process, while HDL is “good” and plays a protective role. In order to approach the effect of statin concentration on lowering LDL levels, a series of fractional derivative equations were used. An elevation of total cholesterol in serum is an indicator of the probability of developing an atheromatic plaque. However, there is no clear threshold of LDL dependence on statin medication for all patients [17,18].

In this context, the object of our study was to develop a more precise understanding of the fundamental role of statins in reducing LDL levels and, indirectly, atheromatic plaque formation. With this as our aim, we included the ratios of [LDL]/[HDL] received from the clinical histories of the patients. Moreover, in the mathematical model, we focused on MDA, one of the main end products resulting from lipid peroxidation due to interaction with free radicals caused by oxidative stress [4,19]. As was mentioned, MDA gives a characteristic absorption band in FTIR spectra that appears at the frequency of 1744 cm^−1^, which originates from the stretching vibration of the functional group *v*CHO [4]. The energy of the aldehydic HC=O chemical bond absorption at the vibrational frequency of 1744 cm^−1^ is equal to 4.98 kcal/mol (Table 1).

### 3.4. Fractional Calculus (FC)

The appropriate way of expressing the mathematical models is to start with the FC computation of classical derivatives and integrals in any arbitrary order, i.e., complex and rational [20]. The advantage of FC over Classical Calculus (CC) is that FC contains the parameter of fractional order, which is necessary for the study of non-local or non-linear phenomena, such as biological systems. Contrasting the fact that CC is uniquely determined, like the calculation of the slope of a band at a single point, FC, through fractional order, manages to consider a history of previous states, such as the clinical effects experienced by the patients. The two most used fractional derivatives (FDs) for continuous systems are the Riemann–Liouville and Caputo FDs.

### 3.5. Caputo Derivative

Caputo fractional derivatives contain a higher fractional order degree to interpret the complex phenomena of biological systems under the multifunctional parameters of the disease or the medication of the patients. The Caputo Fractional Operator (C) is given by Equation (1):

(1) DαxγCft=1Γn−γ∫0tfnst−sγ−n+1ds

where the operator (C) is of the nth order and n is a positive integer, which is the order of the differentiable function f and t(time) is a variable that belongs to the interval (a,x), where a and x are the start and end points, respectively, and Γ is the Gamma–Euler function.

There is a wide range of ordinary differential equation (ODE) models that have been used to capture the mechanism of atherosclerosis. Until now, no ODE has managed to describe the anti-inflammatory and antioxidant role of statins on lipid regulation, as they do not contain the parameter of fractional order, which could be used as an indicator of statin concentration. Therefore, in the present work, we make an effort to propose a mathematical model based only on FC. Thus, our new proposed FDE model contains the correlation between the serum lipoprotein concentrations of HDL and the oxidized cholesterol LDL, which is described by the following set of FDEs:

(2) DαxγCHDLt=βHDLtLDLt

The parameter β satisfies the accumulation of atheromatic plaque components due to oxidative stress caused by disease. The heterogeneous plaque products consist of MDA and phosphorylated, glycosylated (AGEs, advanced glycation end products), and calcified tissues, as detected using FTIR spectroscopy and in accordance with the clinical and literature data [4,18].

However, Equation (2) does not include the free energy of the chemical bonds of the characteristic functional groups produced upon the peroxidation of LDL. To access the present non-local dynamic system of the disease, oxidative stress must be involved. The following Equation (3) allows computing the rate of oxidative stress O_x_ as a function of the time.

(3) DαxγCOxt=−θβHDLtOxt−Oxt

The combination of Equations (2) and (3) finally leads to Equation (4):

(4) DαxγCLDLt=1−θβLDLt+Oxt

Equations (2)–(4) do not involve the energy of the produced specific functional chemical groups of the toxic products caused by the disease. The bond length or energy of the chemical bond is calculated from the vibrational frequencies.

(5) $$E=D\left(1-e^{\tilde{v}{\left(r-r_{0}\right)}^{2}}\right)$$

where D is the bond dissociation energy, and $\tilde{v}$ = $\sqrt{k/2D}$, where $\tilde{v}$ is the vibrational frequency, k is the force constant of the stretching bond between two atoms, r_o_ is the bond length, and r represents any distance between the two atoms.

Furthermore, the parameter α represents the energy of the aldehydes carbonyl bond (*v*C=O, CHO) in 1744 cm^−1^ wavenumbers, which corresponds to 4.98 kcal/mol, the constant parameter which could be considered as a function of the percentage of stenosis.

### 3.6. The Numerical Scheme

To numerically approximate the solution of Equations (2)–(4), we solve our fractional differential equations by using a finite difference method [21]. In order to minimize the computing time, we used a modified form of the Predictor–Corrector method. It is worth noting that the Predictor–Corrector method was initially used by Choi et al. [22]. The PECE method was applied to Caputo fractional differential equations of the following form:

(6) DαxγCyt=ft,yt

The PECE method is primarily based on the trapezoidal quadrature formula. At first, we assumed that our general function y(t) was continuous on an interval of the form [0,T]. For a finite difference numerical approximation scheme, we defined the corresponding step sequentially as t = nk, a positive integer, and k denotes the size of our step. Using induction, we used the following integral form based on the fundamental theorem of calculus:

(7) $$y\left(t_{n+1}\right)=y\left(t_{n}\right)+\int_{t_{n}}^{t_{n+1}}f\left(s,y\left(s\right)\right)$$

By using the trapezoidal quadrature formula to define our integral term, we reach the following equation:

(8) $$\int_{a}^{b}f\left(s\right)ds=\frac{b-a}{2}\left(f\left(a\right)+f\left(b\right)\right)$$

Equation (7) leads to the following form (9):

(9) $$y\left(t_{n+1}\right)=y\left(t_{n}\right)+\frac{k}{2}\left(f\left(t_{n}\right),y\left(t_{n}\right)\right)+f\left(t_{n+1},y\left(t_{\left(n+1\right)}\right)\right)$$

Using the implicit one-step Adams–Bashford–Moulton method, replacing $y\left(t_{n}\right),y\left(t_{n+1}\right)$, we recursively obtain Equation (10):

(10) $$y_{n+1}=y_{n}+\frac{k}{2}\left(f\left(t_{n,}y_{n}\right)+f\left(t_{n+1},\;y_{n+1}\right)\right)$$

Although it seems impossible to solve the equation, since the function f(t,y(t)) is non-linear, we can modify the initial conditions of the application. At this point, we similarly introduced the predictor value, replacing the trapezoidal quadrature formula with the rectangular rule that turns into the well-known Euler Method:

(11) $$y_{n+1}^{p}=y_{n}+kf\left(t_{n},y_{n}\right)$$

Equation (11) leads to the final numerical Equation (12):

(12) $$y_{n+1}=y_{n}+\frac{k}{2}\left(f\left(t_{n,}y_{n}\right)+f\left(t_{n+1},y_{n+1}^{p}\right)\right)$$

**Figure 1:**
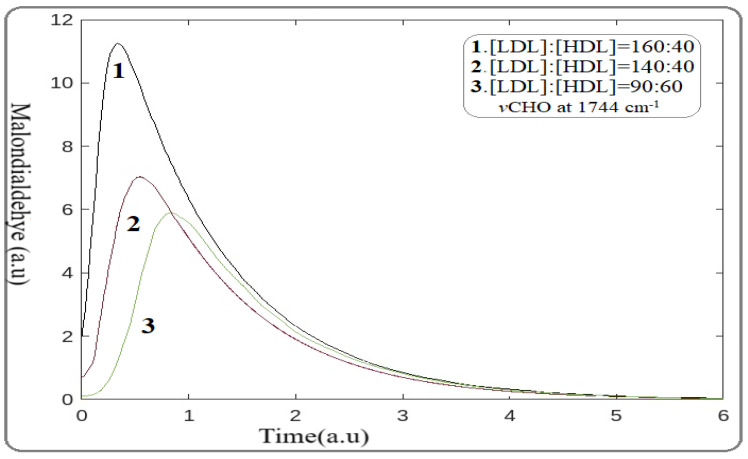
Illustration plot of statin effect on malondialdehyde (MDA) reduction associated with time. Curves 1, 2, and 3 correspond to patients with [LDL]:[HDL] ratios of 160:50, 140:50, and 90:60, respectively. (Wavenumber 1744 cm^−1^, energy 4.98 kcal/mol).

The distorted Equation (2) was applied in our atherosclerotic arterial system. We suggested that the observed changes in the spectra were induced by LDL levels lowering as a result of statin medication. For the calculation, the mathematical package of MATLAB R2024a [13] was applied for the ratios of [LDL]:[HDL] received from blood analysis. The frequency of the sensitive FTIR spectral stretching absorption band of MDA at 1744 cm^−1^ with the energy of 4.98 kcal/mol was used (Table 1). The plotting of MDA production as a function of time in 6 arbitrary units for the distinct [LDL]:[HDL] ratios of 160:50, 140:50, and 90:60, obtained from the medical history of the patients, is shown in Figure 1.

This mathematical model reflects the direct effect of statins on lowering MAD production. Moreover, the observed shift of MAD formation peaks to the right reflects the time delay (hysteresis) in MDA production, suggesting the lowering risk of cardiovascular events. Additionally, these findings provide the antioxidant properties of statins under the present pathophysiological conditions in accordance with the patients’ medical histories, the FTIR spectra, and the literature data [22].

### 3.7. Inorganic Phase of Atherosclerotic Plaque

Coronary mineralization and stenosis is a major risk factor for cardiac disease development. Thus, it is important to study the effect of statins on artery mineralization by using the development model. The mineralized arteries constitute the inorganic phase of an atheromatic plaque. The inorganic phase is promoted by calcium binding preferentially to damaged proteins, enhancing the pathological thickening and sclerosis of the arterial wall. The inorganic phase is composed of calcium carbonate salts (Ca(HCO_3_)_2_ and CaCO_3_), a higher amount of non-biological calcium phosphate (Ca_3_(PO_4_)_2_ and Ca_2_HPO_4_), and biological-like hydroxyapatite. These minerals were detected by using XRD analysis and FTIR spectroscopy, two methods that are sensitive for mineral detection [5,9]. It was suggested that during mineral deposits within arteries, an acidic environment was formed in the patient and that acidosis and anaerobic conditions induce the oxidative hydrolysis of adenosine triphosphate (ATP), which affects calcium homeostasis [23].

**Figure 2:**
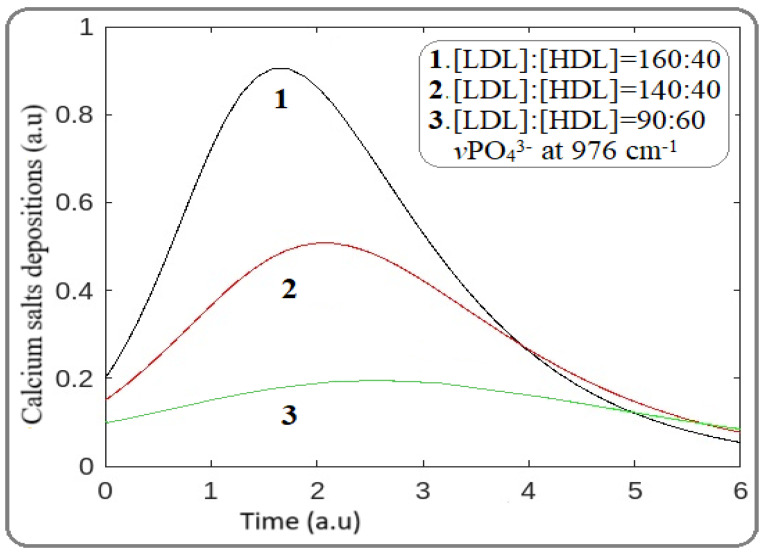
Effect of statins on mineral deposition and atheromatic plaque development. Bands 1, 2, and 3 correspond to [LDL]:[HDL] ratios of 160:40, 140:50, and 90:60. (Wavenumbers 976 cm^−1^, energy 2.79 kcal/mol).

Similar to MAD investigations, for the effect of statins on artery mineralization, the stretching vibration of calcium phosphate groups (*v*PO_4_^3−^) located at wavenumber 976 cm^−1^ with a bond energy of 2.79 kcal/mol was used (Table 1). For the mathematical model, Equations (6)–(12) were once again applied, and the result in Figure 2 shows the preventative effect of statins.

As Figure 2 shows, the decrease in LDL levels in the blood decreases the mineralization of the arteries. Furthermore, the observed shift of the peak to the right suggests a time rate delay in tissue mineralization and atheromatic plaque formation, in accordance with our FTIR spectral data. From the models above, it is demonstrated that statins show a high potential for delaying both lipid oxidation and mineralization.

### 3.8. Calculation of Statin Risk Adverse Point

It is well documented that statins, despite their beneficial effects, cause adverse effects on skeletal muscle, liver, and kidney in some patients [22,23]. Therefore, in this section, our mathematical model must be modified to include parameters that could describe the side effects of statins. In our calculations, a higher reduction in LDL levels was observed by increasing the statin dose, as indicated by the reduction in MDA and mineralization reduction shown in Figure 1 and Figure 2. However, the question of whether that statin dose is beneficial in relation to the critical dose of adverse effects remains unclear.

For this particular problem, we supposed that the application of mathematical differential equations could help us develop a dose-dependent treatment. The developed FDE model could not be used as it does not give any information about the side effects that adverse statin concentrations provoke in patients. Moreover, FDE does not contain the free energy of the chemical bonds of functional groups of the secondary products resulting from oxidative stress. In order to confirm the harmful effects of statins, we further modified our previous model, involving new parameters.

Based on the hypotheses that the artery is a tube and the atheromatic plaque formation is given as a function of F = F(x,t) [9,12], the partial Equations (13)–(14) were used in the model evaluating the effect of statin concentration:

(13) $$\partial_{t}F\left(x,t\right)+div\left(uF\right)=k_{1}O_{X}M\left(t,x\right)\in \left[0,L\right]\times \left[0,h\right]$$

(14) $$\partial_{t}w\left(t,x\right)+div\left(uw\right)=0$$

The evolution of the organic and inorganic components of the atheromatic plaque is described using differential Equation (13). The term k_1_ is the rate of LDL formation; Ox is the oxidized products caused by oxidative stress, associated with the inflammation response (e.g., MDA); M is the deposits of the total atheromatic products; L is the artery length (cm); h is the thickness of the plaque; t is the time; and x is the plaque growth length along the direction of the artery. Equation (14) estimates the rate of atheromatic plaque progression in a system w = w(x,t), and it expresses the local incompressibility caused by artery stenosis. The right-hand side of Equation (5) describes the deposition of foam cells and minerals toward the artery with a speed u. The modification of Equations (13)–(14) gives the final Equation (15):

(15) $$\partial_{t}\left(F+w\right)=k_{1}O_{x}M-div\left(\left(F+w\right)u\right)$$

The local incompressibility is given in Equation (16):

(16) $$\partial_{t}\left(F+w\right)=div\left(\left(F+w\right)u\right)=Adiv\left(u\right)$$

The combination of Equations (14) and (16) gives the following final form (17):

(17) $$0=k_{1}O_{x}M-Adiv\left(u\right)=k_{1}O_{x}M-A\partial_{t}\left(u_{t}\right)$$

Supposing that the blood flows in the opposite direction of the native direction, and by integrating parts of (17), we finally obtain

(18) $$u\left(0\right)-u\left(h\right)=\frac{k_{1}}{A}\int_{0}^{h}O_{x}Mdy=-\frac{k_{1}}{Ah}O_{x}M$$

As was mentioned, considering that the velocity at the time $\left(t0\right)\;u\;$= 0 and that $u=\frac{dh}{dt}$, we obtain the following differential equation:

(19) $$u=\frac{dh}{dt}=\frac{k_{1}}{Ah}O_{x}M$$

where h(t) is the rate of arterial width deformation in 2D with respect to time. However, the concentration of statins is not considered in the preceding classical differential equation since the phenomenon can be regarded as non-local and there has not been a single ordinary differential equation (ODE) model proposed.

(20) DαxγCht=k1AhOxM

where DαxγCht is the Caputo Fractional Operator, A is the area of artery lesions, and O_x_ is the peroxidation products linked to inflammation injury due to oxidative stress. The novelty of the new model currently proposed is that it contains the fractional order γε(0,1) as an indicator of statin concentration, which is considered harmful to the patient.

**Figure 3:**
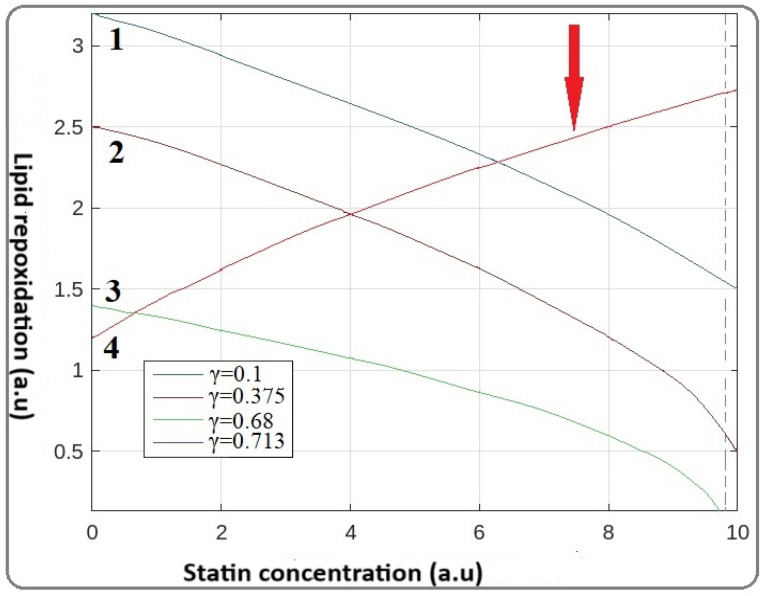
Mathematical modeling of statin correlation between statin dose and lipid peroxidation. Initially, by increasing the concentration of statins (γ parameter), the concentration of lipid peroxides is reduced (curves 1, 2, and 3). For the value of γ = 0.713, the adverse point of statins appears as in curve 4. (Red arrow corresponds to the curve for the reverse -toxic point of statins).

Keeping in mind the reverse behavior of statins, we convert the parameter γ to $\gamma \to \frac{1}{\gamma }$ where the term $\frac{1\;}{\gamma }\;$ is now the quantity that represents the toxic behavior of statins. For the numerical computations of our FDE, the Predictor–Corrector Method (PECE) algorithm was applied. The numerical results of the statin correlation of the various γ values are illustrated in Figure 3.

Figure 3 demonstrates that by increasing the γ value, which corresponds to statin concentration, from 0.1 up to 0.68, the lipid peroxidation is reduced, as was accepted. However, further increasing the factor γ up to 0.713 (Figure 3), does not result in additional LDL level reduction. In contrast, an increased amount of LDL is observed. This pattern supports the suggestion that statins do not protect the patients; there is an optimum administration dose for the patients, and anything in excess of this dose threshold shows adverse effects.

## 4. Discussion

In recent years, metabolic science societies have been focused on formulating guidelines for the treatment of hyperlipidemia, which is one of the most important risk factors for cardiovascular events and even death. Particular effort has gone into reducing LDL levels and developing methods for treatment of excessive LDL levels. Globally, statins are the most recognized drugs for lowering cholesterol levels, following a reduction in cardiovascular events and mortality [1,2,3]. Statins, in addition to cholesterol reduction, also have a wide range of benefits regarding their anti-inflammatory, antioxidant, and fibrinolytic properties [23,24,25]. In the current study, our developed mathematical model, in combination with FTIR spectral data, shows the role statins play in lowering MAD production and artery mineralization.

In vivo and in vitro studies indicate that reactive oxygen species (ROS) are involved in the pathways of artery disease development [3,4,5,6]. Hydroxyl radicals (HO), molecular oxygen (O-O, biradical), and superoxide anions (O_2_^−^) are the most reactive species. The mechanism of artery atherogenesis initiates with lipid peroxidation. The lipid radicals (L·) are produced from the reactions between HO and polyunsaturated fatty acids (PUFA = L) via their addition to double bonds or via the abstraction of hydrogen atoms (H). The derived lipid radicals (L) react with reactive molecular oxygen or oxygen anion (O_2_^−^) radicals [5,6] and generate lipid peroxyl radicals (LOO) and their anions (LOO^−^), among which LDL and MAD end products are considered the most toxic metabolites.

The band intensities in Figure 1 demonstrate that MAD formation is reduced by using statins. This observation indicates that in the primary processes of lipid peroxidation, statins act as scavengers of free radicals originating from redox reactions following inhibition of the lipid peroxidation. This hypothesis is supported by the obtained shift of the peak of MDA production to the right (longer time) by increasing statin dose, which is associated with a delay in lipid peroxidation [26]. These findings point out the anti-inflammatory properties of statins, in agreement with FTIR spectra and clinical data [27]. The molecular structure of statins contains high electron density sites resulting from the double bonds and phenyl rings. Statins react with a diffusion control rate affinity with the high electrophilic HO· radicals, leading to the generation of statin free radicals (S). Via this redox activity, the statins trap free radicals and protect the polyunsaturated membrane lipids from peroxidation, following the reduction in LDL levels. Our suggestion that statins act as free radical scavengers is enhanced by the observed protective role of statins on cardiac muscle after irradiation [28]. Furthermore, the generated statin radicals S· are very reactive species, leading to the formation of various peroxides and fibrotic intermediate products, dependent on the clinical situation of patients. Radiolysis of rosuvastatin gave a number of degradation products which could lead to side effects [29,30,31]. Excess generation of byproducts may modify particular biochemical changes associated with signaling events, leading to side effects.

A significant relationship between statins and the calcification of arteries resulted from the shift of the peak to the right (Figure 2). This occurs because, during atherogenesis, the anaerobic conditions induce oxidative hydrolysis of ATP, affecting calcium homeostasis, which is in agreement with spectroscopic data. It is also important to note that the advanced mathematical model we developed proves that atherogenesis initiates via organic product deposition, and there is a resulting delay in artery mineralization, as shown in Figure 1 and Figure 2.

The advanced mathematical model we developed in the present study demonstrates that the observed clinical side effects of statins are dose-dependent, as illustrated in Figure 3. This suggestion is supported by the observation of Fujita et al., who found that the dose dependency of pleiotropic effects of atorvastatin using the atherosclerotic marker 8-OHdG (8-hydroxyguanosine) was a result of DNA oxidative damage [26]. It is well established that 8-OHdG is produced after the addition of OH radicals in the N7 = C6 double bond of the guanine molecule and is analogous to the degree of lipid peroxidation [32]. Evidence of the involvement of lipid peroxidation byproducts in the non-enzymatic production of F2-isoprostane (F2-Isops) biomarkers also evidences atherosclerosis progression [33,34,35,36]. Moreover, there is evidence that statins act as scavengers of free radicals, inhibiting lipid peroxidation and upregulating the auto-defense system. From this observation, we suggest that statins protect the patient more in the early stages of atherogenesis. It appears that there is a relationship between the alteration of both statin and lipid intermediate secondary products, and side effects. These findings support the hypothesis that an elevated number of byproducts trigger other pathways of immune response, affecting the atheroprotective role of statins [34,35]. However, there are not enough data about the independent action of any secondary lipid and statin intermediates caused by oxidative stress or the great impact they have on other organs that induce side effects in patients. The present mathematical model emphasizes the importance of further systematic pharmacokinetic evaluation of the statin threshold dose to eliminate side effects and optimize LDL therapy.

## 5. Conclusions

The newly developed mathematical model herein described demonstrated that statins are effective drugs for preventing the development of LDL and reducing the risk of cardiovascular events. The beneficial actions of statins were found to be dose-dependent, and there is an optimum dose for statins beyond which side effects are observed. It is suggested that the adverse effects of statins are associated with the elevation of secondary oxidation products. These observations illuminate the clinical pleiotropic properties of statins, which act as antioxidant and anti-inflammatory agents. Such effects of the statins were provided by their delay of atheromatic plaque formation.

## Figures and Tables

**Table 1:** Characteristic FTIR spectral absorption bands (cm^−1^) of coronary arteries, assignments, and energies [4,9].

Wavenumbers cm^−1^	Assignments	Energy kcal/mol
1744	Aldehydes (CHO)—peroxidation product	4.98
1685	β-sheet, anti-//	4.82
1657	Amide I α-helix	4.74
1631	Amide I, random coil, β-sheet-//	4.66
1541	Amide II, α-helix	4.4
1511	β-sheet, parallel	4.32
1163	PO_2^−^_ of phospholipids	3.32
1113	*v*C-O-C, Glycosylation (AGEs)	3.18
976	*v*PO_4_^3−^	2.79

## Data Availability

The data presented in this study are available upon request from the corresponding author.

## Abbreviations

The following abbreviations are used in this manuscript:

8-HOdG	8-hydroxy-2′-deoxyguanosine
AGE	Advanced Glycation End
ATR	Attenuated Total Reflectance
ATP	Adenosine Triphosphate
C	Caputo
CC	Classical Calculus
FC	Fractional Calculus
FD	Fractional Derivatives
FDE	Fractional Differential Equations
FTIR	Fourier-Transform Infrared spectroscopy
HDL	High-Density Lipoprotein
L	Lipids
LDL	Low-Density Lipoprotein
MDA	Malondialdehyde
ODE	Ordinary Differential Equations
Ox	Oxidative Stress
PECE	Predictor–Corrector Method
XRD	X Ray Diffraction
